# Response to the Letter to the Editor by Harris

**DOI:** 10.1186/s13071-019-3439-2

**Published:** 2019-04-24

**Authors:** Telleasha L. Greay, Alireza Zahedi, Anna-Sheree Krige, Jadyn M. Owens, Robert L. Rees, Una M. Ryan, Charlotte L. Oskam, Peter J. Irwin

**Affiliations:** 1 Vector and Waterborne Pathogens Research Group, College of Science, Health, Engineering and Education, Perth, WA Australia; 2 Western Australian State Agricultural Biotechnology Centre, Murdoch University, Perth, WA Australia; 3 Faculty of Veterinary and Agricultural Sciences, The University of Melbourne, Melbourne, VIC Australia

## Abstract

In a letter to the Editor, Harris considers the eight new species of Apicomplexa that were recently identified and named to be invalid on the basis that only molecular characters were provided in the species descriptions. In this response, we counter that the species names are valid as the descriptions have met the requirements of the *International Code of Zoological Nomenclature*; molecular characters can be used to satisfy article 13.1.1 of the code.

**Electronic supplementary material:**

The online version of this article (10.1186/s13071-019-3439-2) contains supplementary material, which is available to authorized users.

## Letter to the Editor

We recently described eight novel apicomplexan species in ticks collected from companion animals in Australia: *Babesia lohae*, *Babesia mackerrasorum*, *Hepatozoon banethi*, *Hepatozoon ewingi*, *Theileria apogeana*, *Theileria palmeri*, *Theileria paparinii* and *Theileria worthingtonorum* [1]. The ticks were screened for *18S* rDNA (*18S*) of *Babesia*, *Hepatozoon* and *Theileria* species using PCR and Sanger sequencing. Further molecular characterisation was performed with near full-length *18S* sequences obtained (~ 1400–1700 bp). The genetic distances showed that the *18S* sequences of the novel species were sufficiently different from their most closely related named species (1.7−7.9% dissimilarity), which warranted new species classifications, in our opinion [1].

In a Letter to the Editor, Harris [2] suggests that specific characters and valid descriptions were not provided for the novel Australian Apicomplexa described in our study [1], and that the proposed names are invalid. Harris [2] disputes the validity of our approach, stating that naming of these parasites based on genetic distinctiveness and phylogenetic relationships, rather than determination of specific characters, was not consistent with the *International Code of Zoological Nomenclature* (ICZN).

The ICZN does not explicitly rule out the use of DNA sequences alone, which are character-based, to describe a new species and does not specifically state that the description should be morphological. Article 13.1.1 states that “To be available, every new name published after 1930 … must be accompanied by a description or definition that states in words characters that are purported to differentiate the taxon” [3].

Traditionally, taxonomists have described organisms using morphological characters, geographical distribution patterns and host specificities. Harris [2] highlights that there is a “Linnean Shortfall” for Apicomplexa, and that molecular studies can help to overcome this issue. Indeed, we are now in an era where it is faster to describe molecular features of microorganisms than morphological characters due to advanced molecular screening approaches. For example, next-generation sequencing enables microorganisms, including viruses, bacteria and protozoans, to be rapidly screened with high accuracy, and millions of sequences from thousands of different species can be generated in a single sequencing assay [4]. The codes and committees governing nomenclature of viral (*International Committee on Taxonomy of Viruses*) and bacterial (*International Code of Nomenclature of Prokaryotes*) microorganisms have largely adopted the use of sequence data to describe novel species, albeit maintaining the outdated requirement of “*Candidatus*” usage for uncultured bacterial species. The criticisms of DNA-sequence-data-only descriptions for protozoans and other eukaryotic taxa have been rebutted in comprehensive reviews and discussions previously [5–7].

The *18S* locus is a conservative marker among eukaryotes and due to the unreliability of morphological characters for delimiting most haemoparasites, it is the most widely used locus to delimit haemoparasite species. Importantly, morphological characters are indistinguishable among many piroplasm and *Hepatozoon* species, especially in their merozoite, trophozoite and gamont stages, and therefore species can be only reliably delimited using molecular characters (e.g. *18S* sequences). Morphological overlaps in piroplasms can also occur at the genus level. For example, *18S* sequence data were used to reclassify *Babesia equi* as *Theileria equi* [8]. *18S* sequence data have been used to identify and classify several previously unknown haemoprotozoans and other eukaryotic parasites in many other studies including, but not limited to, Persing [9], Quick et al. [10], Thomford et al. [11], Herwaldt et al. [12], Katzer et al. [13], Gubbels et al. [14], Nijhof et al. [15], Moss et al. [16], Jorger & Schrodl [7], Ryan et al. [17], Boscaro et al. [18] and Zatti et al. [19].

The *18S* locus evolves more slowly than other barcoding loci, such as the cytochrome *c* oxidase subunit 1 (*cox*1) gene, therefore less variability is observed in *18S* compared with more rapidly evolving genes and can underestimate species diversity in some organisms [20]. The fact that a significant level of variation was observed at the slowly evolving *18S* locus between the novel and known species in our study only strengthens our conclusion that the species are distinct.

It is certainly true that description of additional genetic markers, genomes and morphological features offers a more complete understanding of the characters of a species compared to a single genetic marker. However, as morphology is not always reliable and a comprehensive genetic database of piroplasms and *Hepatozoon* sequences at loci other than the *18S* locus is not available, then it is appropriate to describe the species based on *18S* sequences. There are no requirements under the ICZN about how much or what type of information should be obtained to describe a new taxon, and neither should there be; the types of biological data that scientists can analyse has expanded over the last century, and any type of data that sufficiently delineates novel species can and should be used to describe new organisms. *18S* is the only genetic marker that has been sequenced for *Hepatozoon* species at present. Therefore, *H. banethi* and *H. ewingi* sequence data from other loci would not have assisted us to determine their species status, as there are no other sequences for comparison from other *Hepatozoon* species present in GenBank^®^. As for the piroplasms, only the *18S* gene has been sequenced for all the closest congeners of the new piroplasms described in our study, with one exception; the heat-shock protein gene (*hsp*) has been sequenced for the closest relative to *B. mackerrasorum*, *Babesia macropus* [21]. For all the novel species described in our study, the *18S* sequences were compared to *18S* sequences in GenBank, and the genetic distances between the novel apicomplexans and their closest relatives (described to date) were greater than the genetic distances between the next two most closely related species.

If naming novel microorganisms is constrained by a requirement to describe morphological characteristics, the taxonomic lag (the “Linnean Shortfall”) will be further exacerbated. Taxonomy should enable us to communicate about organisms without confusion, yet there is a propensity to deposit sequence data for novel organisms in GenBank with the “sp.” abbreviation, likely due to the view that morphological descriptions of type-specimens submitted to museums are needed to name species. As a result of this: (i) there are overwhelming numbers of sequences in GenBank that have not been assigned to a species, which makes species identification of similar sequences a cumbersome task (there are currently ~ 3000 *Babesia*, > 2000 *Hepatozoon* and > 2000 *Theileria 18S* sequences on GenBank, and 25% (average length 832 bp), 49% (average length 699 bp) and 27% (average length 927 bp) have no species assigned, respectively); (ii) the ability to communicate about unique and novel “sp.’s” is impeded as there is no requirement for informative isolate names to be provided that are unique to all others; and (iii) there is likely a considerable underestimation of biodiversity on the planet for which data presently exists.

In all cases in our publication, each description was accompanied by GenBank accession numbers of the *18S* sequences, which allows full subsequent comparisons of new and existing sequence data to our sequence data. The text “see above” in the Diagnosis sections of the descriptions refers to the results sections of the paper, where the species differentiations were described and stated in words. The genetic distances were linked to defined characters, the *18S* sequences. The interspecific genetic distances of the *18S* sequences for each novel species that delimit them from other species are stated in the sections “Novel *Babesia* species”, “Novel *Hepatozoon* species”, “Novel *Theileria* species” and “Genetic distance estimates”, where we also refer to summaries of the BLAST results and pairwise genetic distances in Table 5, Additional file 2 and Additional file 4 [1].

At present, there is no standardised format among scientific journals for sequence data descriptions in articles that describe novel species. In the study by Jorger & Schrodl [7], novel species descriptions were accompanied with tables of the nucleotide compositions of the reference DNA sequences, which is not a unique feature of a species, hence GenBank accession numbers of the reference sequences were cited. The nucleotide positions and the genetic distances to other described species are what make the sequences unique. The review by Cook et al. [6] provided a different example of a DNA-sequence-data-only description, with the nucleotide polymorphisms outlined. We have applied this approach to our sequences in Additional file 1: Figure S1 to illustrate why this format was unsuitable for our multiple long sequences, and would also be unsuitable for large datasets (especially genomes). Providing the accession number for the sequences in GenBank and a description of the genetic distances was more concise in our case, and DNA sequences deposited in GenBank convey the same information as sequences in the text of a paper and are freely accessible.

In all cases, we also deposited the type-material (bisected ticks and DNA extracts derived from the other half of the tick) in recognised museum collections at the Australian Centre for Wildlife Genomics at the Australian Museum, the Queensland Museum and the Tasmanian Museum and Art Gallery for future reference.

## Additional file

**Additional file 1: Figure S1.** Nucleotide sequence alignments generated by the MUSCLE alignment tool [22] in Geneious v10.2.2 (http://www.geneious.com, [23]) of the longest reference *18S* sequences for novel *Babesia*, *Hepatozoon* and *Theileria* species described in [1] compared with *18S* sequences from their closest named relatives. Nucleotide polymorphisms are highlighted (adenine (A): red; guanine (G): yellow; thymine (T): green; cytosine (C): blue). Gaps are represented by dashes. Each line is labelled with the species and corresponding GenBank^®^ accession number in parentheses. Nucleotide base positions are numbered above the sequences. **A** *Babesia lohae* (MG593272). **B** *Babesia mackerrasorum* (MG593271). **C** *Hepatozoon banethi* (MG758137). **D** *Hepatozoon ewingi* (MG593275). **E** *Theileria apogeana* (MG758116). **F** *Theileria palmeri* (MG758113). **G** *Theileria paparinii* (MG758115). **H** *Theileria worthingtonorum* (MG758114).
