# Supplementary figures and images for: Response to the Letter to the Editor by Harris
Source: Parasit Vectors. 2019 Apr 24;12:178. doi: 10.1186/s13071-019-3439-2 (PMC6480841; doi:10.1186/s13071-019-3439-2)

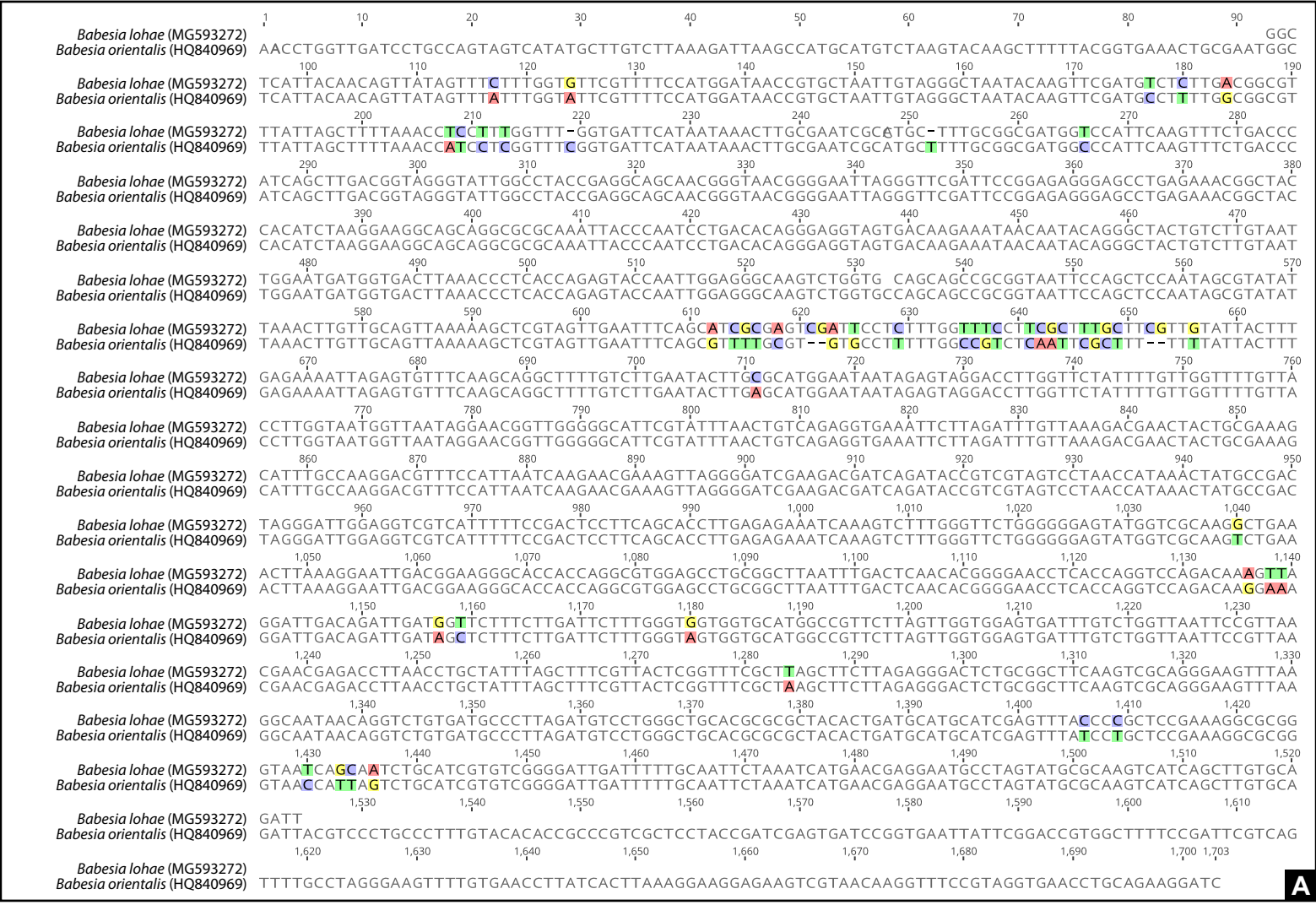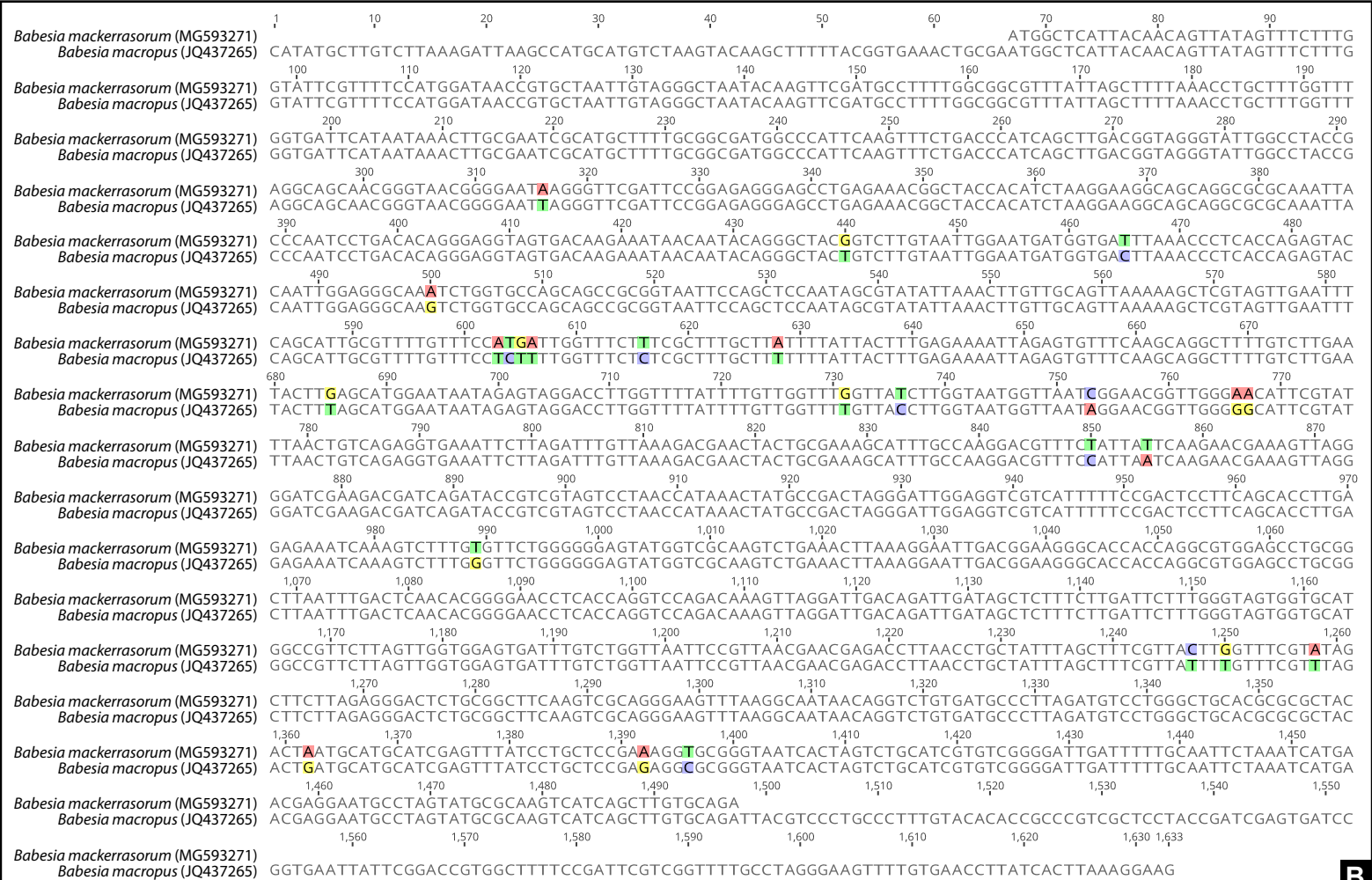

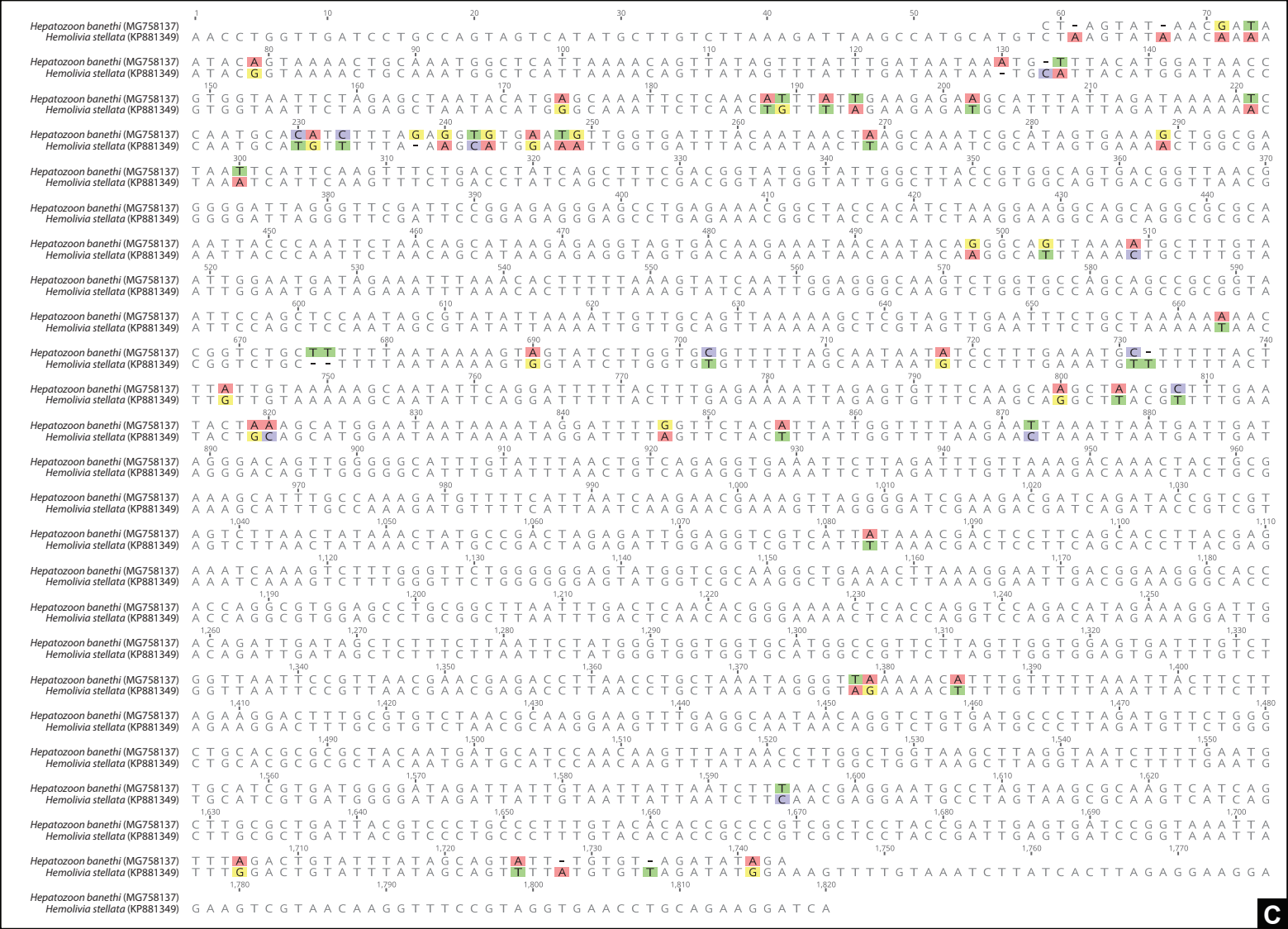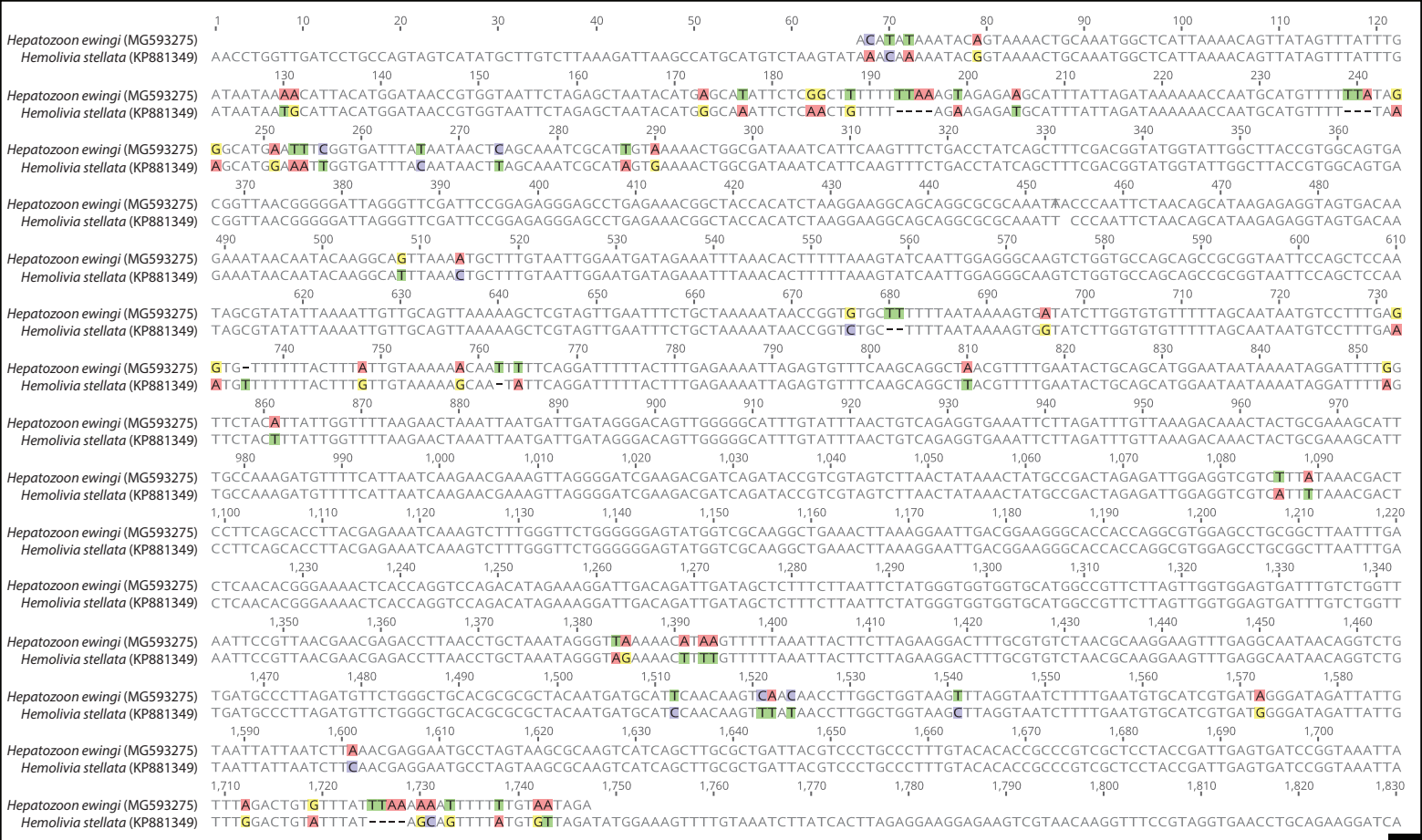

EF

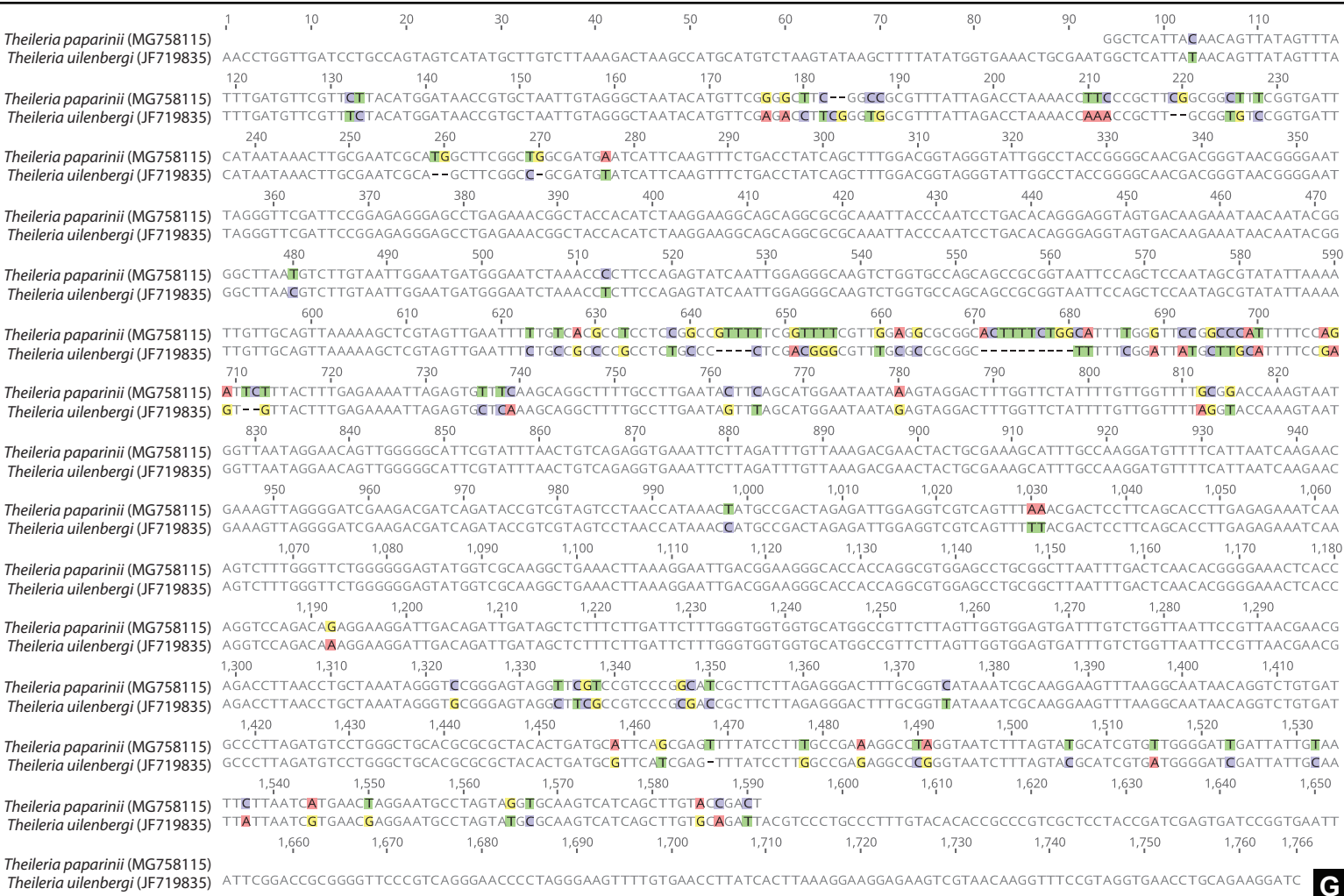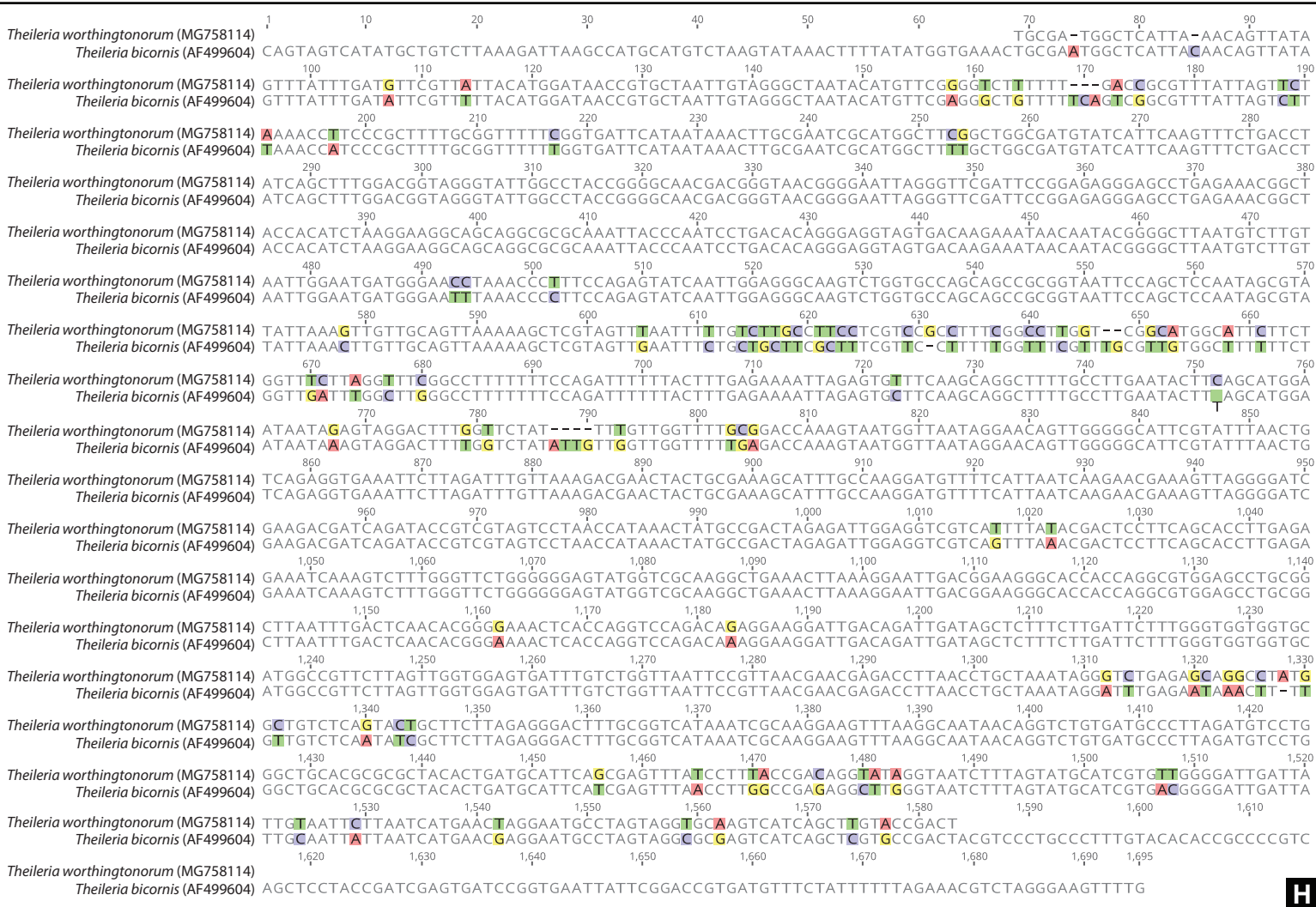

Supplement: Supplementary file 1 — Additional file 1: Figure S1. Nucleotide sequence alignments generated by the MUSCLE alignment tool [22] in Geneious v10.2.2 (http://www.geneious.com, [23]) of the longest reference 18S sequences for novel Babesia, Hepatozoon and Theileria species described in [1] compared with 18S sequences from their closest named relatives. Nucleotide polymorphisms are highlighted (adenine (A): red; guanine (G): yellow; thymine (T): green; cytosine (C): blue). Gaps are represented by dashes. Each line is labelled with the species and corresponding GenBank® accession number in parentheses. Nucleotide base positions are numbered above the sequences. A Babesia lohae (MG593272). B Babesia mackerrasorum (MG593271). C Hepatozoon banethi (MG758137). D Hepatozoon ewingi (MG593275). E Theileria apogeana (MG758116). F Theileria palmeri (MG758113). G Theileria paparinii (MG758115). H Theileria worthingtonorum (MG758114). [file 13071_2019_3439_MOESM1_ESM.pdf]
